# Intralobar Pulmonary Sequestration Presenting as Hemothorax Secondary to Spontaneous Pneumothorax: Case Report and Literature Review

**DOI:** 10.3389/fped.2022.937563

**Published:** 2022-06-30

**Authors:** Tian Chen, Jie Yu, Na Zhang, Chenghao Chen, Libing Fu, Qi Zeng

**Affiliations:** ^1^Department of Thoracic Surgery, Beijing Children’s Hospital, Capital Medical University, National Center for Children’s Health, Beijing, China; ^2^Department of Pathology, Beijing Children’s Hospital, Capital Medical University, National Center for Children’s Health, Beijing, China

**Keywords:** pulmonary sequestration, intralobar, hemothorax, pneumothorax, spontaneous

## Abstract

**Introduction:**

Patients with pulmonary sequestration (PS), a rare congenital lung malformation, are mostly asymptomatic. Recurrent localized infection is a major complication, while sudden hemothorax is extremely rare. We present a case of intralobar PS presenting as hemothorax secondary to spontaneous pneumothorax and comprehensively review the relevant literature.

**Case Report:**

A 16-year-old male presented with chest pain after strenuous exercise. Chest X-ray showed a moderate pneumothorax. After admission and conservative treatment, he developed dizziness, amaurosis, and urinary incontinence. Bedside chest X-ray suggested a massive pleural effusion, and hemothorax was further identified via catheter drainage. Contrast-enhanced computed tomography was performed, and no abnormal blood vessels or leakage of contrast agent were observed. As the hemoglobin level continued to drop, exploratory thoracoscopic surgery was performed immediately. The abnormal systemic artery supplying the lung tissue was found to be ruptured; therefore, ligation of the abnormal artery with resection of the diseased lung tissue was performed. Pathological examination revealed non-specific manifestations of PS. He was followed up for 1 year without related complications.

**Conclusion:**

Our case suggests that the abnormal supply vessels of PS are unstable, which may cause sudden hemothorax. Therefore, patients with PS should undergo surgery promptly after diagnosis. In patients with hemothorax, we should consider the diagnosis of PS; however, contrast-enhanced computed tomography or angiography cannot confirm the diagnosis in all cases. Surgical intervention is recommended in emergency settings.

## Introduction

Pulmonary sequestration (PS), with an incidence of 0.15–1.8%, is a rare congenital lung malformation, manifesting as a non-functional cystic mass of lung tissue supplied by abnormal systemic arteries (1). Most patients with PS are asymptomatic, while the major complication is recurrent localized infection. Symptomatic patients require surgical resection of the diseased lung tissue. However, whether asymptomatic patients require surgical intervention is controversial (2, 3). Hemothorax due to PS has not been reported in retrospective studies from multiple centers (4). We present a case of intralobar PS presenting as hemothorax following an initial spontaneous pneumothorax. Additionally, we discuss the possible causes, clinical manifestations, diagnosis, and management of this rare complication based on a comprehensive literature review and our experiences.

## Case Report

**FIGURE 1:**
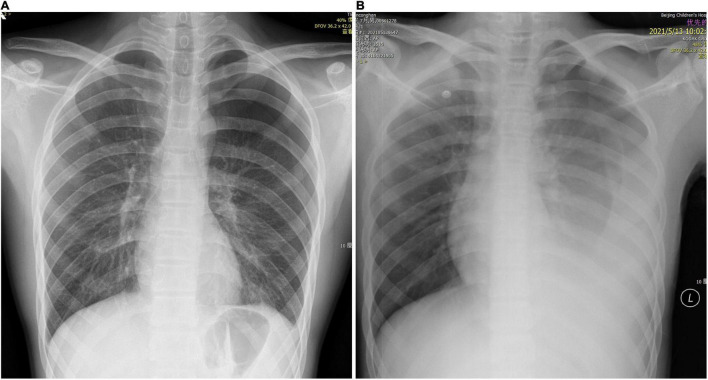
Chest X-ray. **(A)** Left pneumothorax volume of approximately 30%. **(B)** Left pneumothorax with massive pleural effusion.

A 16-year-old male presented to our center with sudden-onset left chest pain after strenuous exercise. For 6 months prior to presentation, he experienced slight left chest pain several times after exercise, which could be relieved by rest. He denied other previous symptoms or comorbidities. On admission, his pain was persistent, severe, prickling-like, with radiation to the back, and was accompanied by mild wheezing and dyspnea. He was of leptosome type and presented with the following: body temperature of 36.7°C, pulse rate of 100 beats/min, respiration rate of 28 beats/min, and blood pressure of 117/73 mmHg. His left lung had diminished breath sounds and tympanic percussion. Hemoglobin and leukocyte levels were 147 g/L and 7.06 × 10^9^/L, respectively. Two chest X-rays 3 h apart showed a pneumothorax volume of approximately 30% and slight dulling of the left costophrenic angle, without tendencies to worsen (Figure 1A).

**FIGURE 2:**
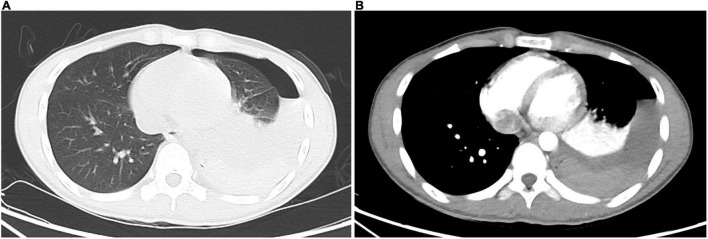
Contrast-enhanced chest computed tomography. **(A)** The lung window shows left hydropneumothorax; dorsal side of left lower lobe shows consolidation, which presents as atelectasis after compression. **(B)** The mediastinal window shows left hydropneumothorax; consolidation of the dorsal side of the left lower lobe is enhanced, which presents as atelectasis after compression; high-density lesion in the left dorsal pleural cavity has no enhancement, which is a blood clot; no blood vessels with abnormal morphology and density or contrast agent leaking into the thoracic cavity are found.

The patient was initially diagnosed with spontaneous pneumothorax. After admission, he was monitored and treated conservatively, which provided pain relief. At the 14th hour after admission, he developed dizziness, amaurosis, and urinary incontinence twice, and the symptoms resolved spontaneously after brief rest. When the symptoms occurred, he had pale lips with profuse sweating, but with good skin elasticity. Blood glucose level was normal. Blood pressure of the two times was 115/64 and 108/60 mmHg, respectively. Bedside chest X-ray suggested left pneumothorax with massive pleural effusion (Figure 1B), and hemoglobin level was 105 g/L. Furthermore, he underwent closed thoracic drainage under local anesthesia, and dark red bloody fluid was drained. After slowly releasing 860 mL of fluid, the drainage tube was clamped. Considering the massive blood loss, he was given intravenous fluids. To clarify its etiology, contrast-enhanced computed tomography (CT) was performed and suggested a high-density lesion in the left dorsal pleural cavity without enhancement, which was considered to be a blood clot. Meanwhile, the dorsal side of the left lower lobe was significantly enhanced, which was considered as consolidation and atelectasis after compression. No large blood vessels with abnormal morphology and density or contrast agent leaking into thoracic cavity were found (Figures 2A,B). Additionally, no intrathoracic space-occupying lesions were found by ultrasonography. At the 21st hour after admission, the hemoglobin level was 105 g/L, while blood biochemistry and coagulation were normal. Considering the stable condition and hemoglobin level of the patient, the patient was closely monitored. At the 27th and 37th hours after admission, hemoglobin levels were 99 and 94 g/L, respectively. However, after transfusing 1 unit of red blood cells and 200 mL plasma, the hemoglobin level dropped to 88 g/L at the 51st hour. As unexplained active bleeding was considered, urgent exploratory thoracoscopic surgery was performed.

**FIGURE 3:**
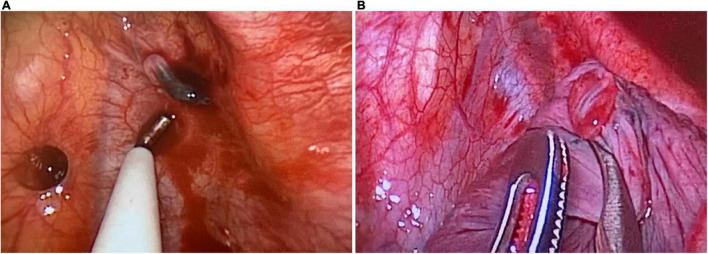
Intraoperative exploration. **(A)** The abnormal branch of the left subclavian artery is ruptured, with a diameter of 2 mm. The proximal end is spasmodic, and the distal end forms thrombus, which is bleeding slowly. **(B)** The abnormal lung tissue in the apical segment of the upper lobe of the left lung, measuring 1 × 1 × 1 cm. The ruptured blood vessel supplying the abnormal lung tissue can be seen, with a diameter of 2 mm.

**FIGURE 4:**
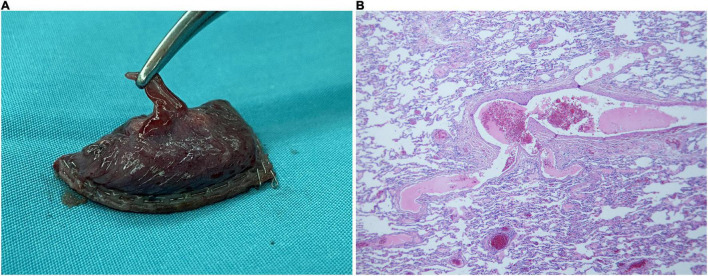
Pathological specimen and microscopic findings. **(A)** The abnormal supply vessel in the upper lobe apical segment of the left lung is ruptured, with a diameter of 2 mm. The surrounding lung tissue erodes and appears red. **(B)** There are many lymphocytic infiltration in the pulmonary interstitium along with mild hyperplasia of the pleural fibrous tissue. Numerous foam cells, cholesterol crystals, and multinucleated giant cell reactions are seen in the alveoli near the pleura.

During intraoperative exploration, 700 mL of old bloody effusion was seen in the pleural cavity wherein blood clots were found. An abnormal branch from the left subclavian artery at the top of the thoracic cavity was ruptured, and the ruptured end was 2 mm in diameter. The proximal end was spasmodic, and the distal end formed a thrombus, which was bleeding slowly (Figure 3A). Meanwhile, abnormal lung tissue, measuring 1 × 1 × 1 cm in the apical segment of the upper lobe of the left lung, eroded and appeared red. A ruptured blood vessel supplying the abnormal lung tissue could be seen, with a diameter of 2 mm (Figures 3B, 4A). The abnormal blood vessel, arising from the left subclavian artery and suppling the abnormal lung tissue, ruptured and caused the hemothorax. Finally, the ruptured blood vessel from the abnormal branch was ligated, and the abnormal lung tissue in the apical segment was resected.

Microscopic examination of the specimen revealed lymphocytic infiltration in the pulmonary interstitium and mild hyperplasia of the pleural fibrous tissue. Numerous foam cells, cholesterol crystals, and multinucleated giant cell reactions were seen in the alveoli near the pleura (Figure 4B). These non-specific manifestations were considered as emphysema and endogenous lipoid pneumonia secondary to sequestered lung tissue.

The hemoglobin level and chest X-ray findings returned to normal after surgery, and the patient was discharged on the 6th day of hospitalization after removing the chest drainage tube. He was followed up for 1 year without related complications.

## Discussion

Pulmonary sequestration was first named by Pryce in 1946. It is considered when the lung bud is separated from the gastrula in early embryonic development and the connection between the splanchnic vascular plexus surrounding the lung bud and dorsal aorta is absorbed incompletely, forming abnormal branching blood vessels of the aorta that pull, separate, and supply the sequestered lung tissue (5). The sequestered lung tissue is cystic and without oxygenation function. It is supplied by abnormal branches of systemic arteries and is generally not in communication with the normal bronchial tree. According to the presence or absence of separate visceral pleura, PS is divided into the intralobar and extralobar types (6).

Intralobar PS was first described by Huber in 1777 (6), accounting for approximately 75% of PS. Intralobar sequestered lung tissue and adjacent lobe are covered by the same visceral pleura, which may form communication with the normal bronchial tree. In most cases, the sequestered lung tissue is located in the posterior basal segment of the left lower lobe. Its arterial blood supply mainly comes from the thoracic and abdominal aorta, and its venous blood mainly flows back to the pulmonary vein (7).

Extralobar PS was first described by Rokitansky and Rektorzik in 1861 (6). Extralobar sequestered lung tissue is covered by a separate visceral pleura outside the normal lobe, which has no communication with the normal bronchial tree. The sequestered lung tissue is mostly located between the left lung base and diaphragm, or in the abdominal cavity, diaphragm, mediastinum, etc. Its arterial blood supply mainly comes from the thoracic and abdominal aorta, and its venous blood mainly flows back to the azygos and hemiazygos veins. However, the supplying arteries usually have several variations and smaller diameters. Additionally, extralobar PS is often combined with diaphragmatic hernia, pulmonary cystic disease, bronchopulmonary foregut malformation, and other diseases (1, 7).

The sequestered lung tissue and normal bronchial tree may form a connection for some reason, which can cause recurrent lung infection. Normal pulmonary arteriovenous atrophy and abnormal systemic arterial supply can cause a blood shunt. A left-to-left shunt caused by a systemic artery-pulmonary vein shunt produces symptoms of congestive heart failure, and a left-to-right shunt caused by a systemic artery-azygos vein shunt produces symptoms of hypoxia (6). Generally, intralobar sequestered lung tissue can communicate with the normal bronchial tree; hence, patients can present with cough, sputum, fever, and other signs of infection. Infection can invade the pulmonary interstitium and cause bloody infiltration of the alveoli, leading to hemoptysis. Infection also can spread to the pleura, leading to chest pain and exudative pleural effusion. However, extralobar sequestered lung tissue has no communication with the normal bronchial tree; hence, patients usually have no obvious symptoms (1).

A preoperative diagnosis of PS mainly depends on prenatal ultrasound, angiography, contrast-enhanced CT, or magnetic resonance angiography (MRA) to find the abnormal supply arteries. Surgical intervention requires identification and ligation of the abnormal supply vessels and resection of the sequestered lung tissue. Thoracoscopic surgery is preferred over thoracotomy or vascular embolization (8, 9).

Hemothorax is a rare, urgent, serious, and misleading clinical manifestation of PS. The PubMed database was searched with the keyword “pulmonary sequestration and hemothorax,” and 25 related cases were retrieved. Unfortunately, two cases had incomplete information (10, 11). Finally, 13 patients with intralobar PS and 10 patients with extralobar PS presenting as hemothorax were reviewed.

A total of 13 cases of intralobar PS manifested as hemothorax, with a median onset age of 30 years and a predilection for males. Initial symptoms mainly included chest pain (11/13), hemoptysis (11/13), and respiratory discomfort (7/13); seldom accompanied by fever (4/13) or elevated carcinoembryonic antigen (CEA) in pleural effusion (1/13); and up to 7 patients developed shock. CT examination mainly showed intrapulmonary mass (10/13), pleural effusion (9/13), and pulmonary consolidation (2/11). It should be noted that after confirming pleural effusion to be bloody by puncture or catheter drainage, five patients underwent further contrast-enhanced CT examination. Abnormal supply arteries were observed in four patients, two of which showed concentrated mass-like enhancement focus, while no patient had contrast agent leakage into the thoracic cavity. Additionally, four patients underwent further arteriography, and abnormal supply arteries were observed in all cases. The final diagnosis mainly depended on vascular imaging (8/13) or surgical exploration (4/13).

The causes of hemothorax in intralobar PS were analyzed along with intraoperative condition and pathological examination. Rupture or obstruction of the cystic sequestered lung tissue and abnormal supply vessels were reported in all cases, while arterial thrombosis, fibrinoid necrosis, arteritis, inflammatory necrosis, and hemorrhage of lung tissue could be observed microscopically. This might be due to the invasive effect of Aspergillus infection, or changes of state in blood vessel and blood flow caused by hypertension, aneurysm, salicylic acid drugs, and mechanical compression of the pectus excavatum. An additional excel file shows the details of these cases (see Supplementary Material 1) (12–24).

A total of 10 cases of extralobar PS manifested as hemothorax, with a median onset age of 33 years and a predilection for males. Initial symptoms mainly included chest pain (9/10) and respiratory discomfort (8/10); seldom accompanied by fever (3/10) or hemoptysis (1/10); and only one patient developed shock. CT examination mainly showed pleural effusion (10/10), extrapulmonary mass (8/10), and pulmonary compression and consolidation (5/10). After confirming a bloody pleural effusion, four patients underwent further contrast-enhanced CT examination. No abnormal supply arteries were found, two of which showed concentrated mass-like enhancement focus, and one patient had contrast agent leakage into the thoracic cavity. Additionally, two patients underwent further arteriography, one of whom presented with an abnormal supply artery, while the other patient did not. The final diagnosis mainly depended on surgical exploration (7/10) or pathological examination (2/10).

The causes of hemothorax in extralobar PS were analyzed. Mechanical torsion or rupture of the extrapulmonary part of the abnormal supply vessels was reported in two cases, while hemorrhage and necrosis of the lung tissue could be observed microscopically. This might be because pregnancy, cough, and tumor changed the spatial structure of the pleural cavity. In contrast, obstruction or rupture of the abnormal supply vessel inside the sequestered lung tissue was reported in other cases, while vascular occlusion, hemorrhage, and necrosis of the lung tissue could be found microscopically. This might be because smaller blood vessel diameter, aneurysm, and anticoagulant treatment affected the state of the blood vessel and blood flow. An additional excel file shows the details of these cases (see Supplementary Material 2) (25–33).

Pulmonary sequestration is a predisposing factor of hemothorax. First, the pressure from the abnormal systemic artery is higher; however, it forms a direct supply to the artery of the sequestered lung tissue. Second, from the center to the periphery of the abnormal supply artery, there is a change from muscular to elastic artery. Abnormal supply artery with elastic component is similar to pulmonary artery, which is relatively fragile (34, 35). Third, most of the abnormal supply vessels have atherosclerosis, occlusive endarteritis, or venosclerosis (36). Although angiosclerosis is associated with age, these changes in the abnormal supply vessels can also be found in the pediatric population, which further confirms the vulnerability of the abnormal supply vessels (37).

Our patient initially developed spontaneous pneumothorax, which caused apparent separation of the visceral and parietal pleura. Afterward, the abnormal supply vessel exposed in the pleural cavity was pulled and ruptured, resulting in a hemothorax. Non-traumatic hemothorax can be secondary to malignancy, vascular malformation, pulmonary infarction or hemorrhage, coagulation disorder, aortic aneurysm or dissection, infection, endometriosis, rib exophytic osteosarcoma, and other diseases (38). When patients develop symptoms of chest pain, respiratory discomfort, and hemoptysis, and CT examination shows pleural effusion and mass, especially a cystic mass located in the left inferior pulmonary ligament, we must consider a diagnosis of hemothorax caused by PS. In this case, after hemothorax was confirmed by catheter drainage, the patient underwent contrast-enhanced CT examination. However, no abnormal blood vessel and contrast agent leaking into the thoracic cavity was found. This might be due to spasm and thrombosis after rupture of the abnormal supply vessel and the reduction of negative pressure in the pleural cavity after pneumothorax, leading to reduced blood leakage. Meanwhile, the diameter of the abnormal supply vessel was smaller and the bleeding was slow, which could not be displayed in the time window of the contrast-enhanced CT. Therefore, a diagnosis of PS cannot be completely ruled out when the abnormal supply artery is not definitely displayed by contrast-enhanced CT or arteriography.

Similar to most cases in the literature, after the identification and ligation of the abnormal blood vessel and resection of the abnormal lung tissue through thoracoscopy, our patient had a good prognosis. However, it cannot be denied that shock may occur after hemothorax due to PS; hence, untimely diagnosis and treatment may even lead to death (20). There are numerous patients with PS who do not undergo surgical intervention or are not diagnosed for many years (1, 6, 37). Since the abnormal supply vessel is a destabilizing factor for the occurrence of hemothorax in PS patients, we suggest that patients with PS should undergo surgery regardless of symptoms. In cases of sudden hemothorax due to PS, vascular embolization can effectively block the abnormal supply vessels (16); however, simple vascular pedicle embolization or ligation will further cause infection of the residual sequestered lung tissue. Hence, surgical intervention is necessary and recommended. Conversion from thoracoscopic surgery to thoracotomy may be required in complex or emergency situations (17, 21, 24).

## Conclusion

We report a case of intralobar PS presenting as hemothorax following an initial spontaneous pneumothorax. The risk of surgery should be weighed against the risk of complications attributed to PS. Since an abnormal supply vessel is a destabilizing factor for hemothorax in patients with PS, we suggest these patients should undergo surgery in time after diagnosis. For patients with hemothorax, the diagnosis of PS needs to be considered; however, contrast-enhanced CT or angiography cannot confirm the diagnosis in all cases. The final diagnosis may depend on surgical exploration or pathological examination. Surgery is a necessary and recommended intervention for cases with hemothorax due to PS.

## Data Availability Statement

The original contributions presented in this study are included in the article/Supplementary Material, further inquiries can be directed to the corresponding author.

## Ethics Statement

Written informed consent was obtained from the minor(s)’ legal guardian/next of kin for the publication of any potentially identifiable images or data included in this article.

## Author Contributions

TC analyzed the case, designed the content, and wrote the manuscript. JY and NZ managed the patient, interpreted the details, and revised the manuscript. CC performed the surgery and interpreted the details. LF performed the pathological diagnosis. QZ performed the critical revision for intellectual content. All authors have read and approved the final manuscript.

## Conflict of Interest

The authors declare that the research was conducted in the absence of any commercial or financial relationships that could be construed as a potential conflict of interest.

## Publisher’s Note

All claims expressed in this article are solely those of the authors and do not necessarily represent those of their affiliated organizations, or those of the publisher, the editors and the reviewers. Any product that may be evaluated in this article, or claim that may be made by its manufacturer, is not guaranteed or endorsed by the publisher.

## Abbreviations

PS: pulmonary sequestration
CT: computed tomography
MRA: magnetic resonance angiography
CEA: carcinoembryonic antigen.

## References

[1] Corbett HJ Humphrey GM. Pulmonary sequestration. *Paediatr Respir Rev.* (2004) 5:59–68. 10.1016/j.prrv.2003.09.009 15222956

[2] Adzick NS Harrison MR Crombleholme TM Flake AW Howell LJ. Fetal lung lesions: management and outcome. *Am J Obstet Gynecol.* (1998) 179:884–9. 10.1016/s0002-9378(98)70183-8 9790364

[3] Laberge JM Puligandla P Flageole H. Asymptomatic congenital lung malformations. *Semin Pediatr Surg.* (2005) 14:16–33. 10.1053/j.sempedsurg.2004.10.022 15770585

[4] Wei Y Li F. Pulmonary sequestration: a retrospective analysis of 2625 cases in China. *Eur J Cardiothorac Surg.* (2011) 40:e39–42. 10.1016/j.ejcts.2011.01.080 21459605

[5] Pryce DM. Lower accessory pulmonary artery with intralobar sequestration of lung; a report of seven cases. *J Pathol Bacteriol.* (1946) 58:457–67. 20283082

[6] Carter R. Pulmonary sequestration. *Ann Thorac Surg.* (1969) 7:68–88. 10.1016/s0003-4975(10)66147-4 4883836

[7] Smith RAA. Theory of the origin of intralobar sequestration of lung. *Thorax.* (1956) 11:10–24. 10.1136/thx.11.1.10 13311855 PMC1019399

[8] Cho MJ Kim DY Kim SC Kim KS Kim EA Lee BS. Embolization versus surgical resection of pulmonary sequestration: clinical experiences with a thoracoscopic approach. *J Pediatr Surg.* (2012) 47:2228–33. 10.1016/j.jpedsurg.2012.09.013 23217881

[9] Zhang N Zeng Q Chen C Yu J Zhang X. Distribution, diagnosis, and treatment of pulmonary sequestration: report of 208 cases. *J Pediatr Surg.* (2019) 54:1286–92. 10.1016/j.jpedsurg.2018.08.054 30291025

[10] Beau A Collignon P Guillaumot M Vert P. [Spontaneous hemothorax revealing a pulmonary sequestration. Anatomo-clinical study]. *Ann Chir Infant.* (1966) 7:59–62. 5906601

[11] Tabatadze KG Shtern RD Blokhin MK. [A rare complication of intralobar pulmonary sequestration]. *Khirurgiia.* (1991):139–40. 1770724

[12] Oxman LM. Intralobar sequestration causing hemoptysis and hemothorax. *N Y State J Med.* (1974) 74:961–2. 4525983

[13] Laurin S Aronson S Schüller H Henrikson H. Spontaneous hemothorax from bronchopulmonary sequestration. Unusual angiographic and pathologic-anatomic findings. *Pediatr Radiol.* (1980) 10:54–6. 10.1007/bf01644345 7422394

[14] Zapatero J Baamonde C Bellán JM Aragoneses FG Orusco E Perez Gallardo M Hemothorax as rare presentation of intralobar pulmonary sequestration. *Scand J Thorac Cardiovasc Surg.* (1983) 17:177–9. 10.3109/14017438309109885 6612259

[15] Iliás L Pálffy G Szónyi P Taller A. [Massive haemothorax caused by intralobar pulmonary sequestration]. *Orv Hetil.* (1996) 137:1263–5. 8757097

[16] Wandschneider W Illiasch H. Intralobar sequestration: a rare cause of severe hemothorax. *J Thorac Cardiovasc Surg.* (2003) 126:872–3. 10.1016/s0022-5223(03)00692-5 14502174

[17] Hofman FN Pasker HG Speekenbrink RG. Hemoptysis and massive hemothorax as presentation of intralobar sequestration. *Ann Thoracic Surg.* (2005) 80:2343–4. 10.1016/j.athoracsur.2004.06.061 16305904

[18] Tamura Y Kushibe K Tojo T Takahama M Kimura M Taniguchi S. Intralobar sequestration presenting as a large intrapulmonary hematoma and massive hemothorax. *Jpn J Thorac Cardiovasc Surg.* (2006) 54:437–9. 10.1007/s11748-006-0026-1 17087324

[19] Arsalane A Parrot A Assouad J Tchanderli R Bazelly B. [Spontaneous hemothorax: a rare but serious complication of intralobular pulmonary sequestration]. *Rev Pneumol Clin.* (2006) 62:30–3. 10.1016/s0761-8417(06)75410-4 16604038

[20] Wang HW Lu JY Sun JZ Xiao Y Wen B. Massive hemoptysis and hemothorax: a rare but fatal complication of intralobar sequestration. *Chinese Med J.* (2012) 125:2638–40. 22882954

[21] Kleffner T Holzer M Hülskamp G Feindt P Groetzner J. Acute hemoptysis and pulmonary hemorrhage after judo as presentation of intralobar sequestration. *Thorac Cardiovasc Surg.* (2013) 61:172–4. 10.1055/s-0032-1304552 22535674

[22] Yoshitake S Hayashi H Osada H Kawahara M. Emergency laparotomy helped the resection of an intralobar pulmonary sequestration with haemorrhagic shock. *Eur J Cardiothorac Surg.* (2013) 43:190–2. 10.1093/ejcts/ezs394 22764148

[23] Alptekin Erkul GS Erkul S Parlar A Çekirdekçi A. An uncommon cause of massive haemothorax and treatment under cardiopulmonary bypass. *Interact Cardiovasc Thorac Surg.* (2021) 32:996–7. 10.1093/icvts/ivab008 33537705 PMC8691591

[24] Luo W Hu TC Luo L Li YL. Pulmonary sequestration with aspergillus infection presenting as massive hemoptysis and hemothorax with highly elevated carcinoembryonic antigen in pleural effusion that mimics advanced lung malignancy. *Eur J Med Res.* (2021) 26:48. 10.1186/s40001-021-00519-5 34034813 PMC8146657

[25] Klöti J Walliser G Sacher P. [Non-trauma-induced hemothorax–a certain leading symptom of a malignant tumor?]. *Z Kinderchir.* (1989) 44:115–8. 10.1055/s-2008-1043214 2660465

[26] Avishai V Dolev E Weissberg D Zajdel L Priel IE. Extralobar sequestration presenting as massive hemothorax. *Chest.* (1996) 109:843–5. 10.1378/chest.109.3.843 8617101

[27] Guska S. [Hemothorax caused by bleeding inside extralobar pulmonary sequestration in a patient on anticoagulation therapy]. *Med Arh.* (2004) 58:55–8. 15017908

[28] Ghraïri H Zendah I Ammar J Zidi A Kilani T Hamzaoui A. [Abundant hemothorax revealing extralobular pulmonary sequestration]. *Rev Pneumol Clin.* (2006) 62:27–9. 10.1016/s0761-8417(06)75409-8 16604037

[29] Pinto Filho DR Avino AJ Brandão SL. Extralobar pulmonary sequestration with hemothorax secondary to pulmonary infarction. *J Bras Pneumol.* (2009) 35:99–102. 10.1590/s1806-37132009000100015 19219338

[30] Tetsuka K Endo S Kanai Y Yamamoto S. Extralobar pulmonary sequestration presenting as hemothorax. *Interact Cardiovasc Thorac Surg.* (2009) 9:547–8. 10.1510/icvts.2009.209254 19520706

[31] Selvaratnam R Srirangalingam U McLean E Lang-Lazdunski L Goulden P. A rare cause of acute chest pain in a young adult. *Clin Med.* (2011) 11:265–7. 10.7861/clinmedicine.11-3-265 21902082 PMC4953322

[32] Di Crescenzo V Laperuta P Napolitano F Carlomagno C Garzi A Vitale M. Pulmonary sequestration presented as massive left hemothorax and associated with primary lung sarcoma. *BMC Surg.* (2013) 13(Suppl. 2):S34. 10.1186/1471-2482-13-s2-s34 24267748 PMC3851145

[33] Okubo Y Hamakawa H Ueda H Imai Y Takahashi Y. Extralobar sequestration presenting as sudden chest pain due to hemothorax. *Ann Thorac Surg.* (2016) 101:e27. 10.1016/j.athoracsur.2015.10.025 26694308

[34] Gerard FP Lyons HA. Anomalous artery in intralobar bronchopulmonary sequestration; report of two cases demonstrated by angiography. *N Engl J Med.* (1958) 259:662–6. 10.1056/nejm195810022591402 13590421

[35] Turk LN III Lindskog GE. The importance of angiographic diagnosis in intralobar pulmonary sequestration. *J Thorac Cardiovasc Surg.* (1961) 41: 299–305. 13778698

[36] Bergman M Flance IJ. Vascular changes in bronchopulmonary sequestration; observations in two cases with multiple accessory systemic arteries. *J Thorac Surg.* (1956) 31:199–210. 13296091

[37] Savic B Birtel FJ Tholen W Funke HD Knoche R. Lung sequestration: report of seven cases and review of 540 published cases. *Thorax.* (1979) 34:96–101. 10.1136/thx.34.1.96 442005 PMC471015

[38] Martinez FJ Villanueva AG Pickering R Becker FS Smith DR. Spontaneous hemothorax. Report of 6 cases and review of the literature. *Medicine.* (1992) 71:354–68. 1435230

